# High Trait Anxiety: A Challenge for Disrupting Fear Memory Reconsolidation

**DOI:** 10.1371/journal.pone.0075239

**Published:** 2013-11-18

**Authors:** Marieke Soeter, Merel Kindt

**Affiliations:** 1 Department of Clinical Psychology, University of Amsterdam, Amsterdam, The Netherlands; 2 Research Priority Program Brain and Cognition, Cognitive Science Center Amsterdam, University of Amsterdam, Amsterdam, The Netherlands; Universidad de Granada, Spain

## Abstract

Disrupting reconsolidation may be promising in the treatment of anxiety disorders but the fear-reducing effects are thus far solely demonstrated in the average organism. A relevant question is whether disrupting fear memory reconsolidation is less effective in individuals who are vulnerable to develop an anxiety disorder. By collapsing data from six previous human fear conditioning studies we tested whether *trait anxiety* was related to the fear-reducing effects of a pharmacological agent targeting the process of memory reconsolidation - *n* = 107. Testing included different phases across three consecutive days each separated by 24 h. Fear responding was measured by the eye-blink startle reflex. Disrupting the process of fear memory reconsolidation was manipulated by administering the β-adrenergic receptor antagonist *propranolol HCl* either before or after memory retrieval. Trait anxiety uniquely predicted the fear-reducing effects of disrupting memory reconsolidation: the higher the trait anxiety, the less fear reduction. Vulnerable individuals with the propensity to develop anxiety disorders may need higher dosages of propranolol HCl or more retrieval trials for targeting and changing fear memory. Our finding clearly demonstrates that we cannot simply translate observations from fundamental research on fear reduction in the average organism to clinical practice.

## Introduction

Newly formed memories are initially “labile” and susceptible to disruption before being consolidated into stable long-term memories [1]. Ample evidence demonstrates that this consolidation process depends on the synthesis of new proteins [2], [3]. For decades it was thought that after consolidation a memory trace becomes permanent and unmodifiable [4]. But last years have witnessed rapidly emerging evidence for the plasticity of memories. Upon their retrieval consolidated memories may temporarily return into a labile state requiring de novo protein synthesis for restabilization [5], [6]. Interfering with this restabilization process through pharmacological agents is referred to as disrupting reconsolidation and enables the modification of the original memory representation [5], [7]. As a result disrupting reconsolidation may well point to a novel therapeutic strategy providing long-term cure for patients suffering from anxiety disorders. By now a substantial body of animal and human research indeed indicates that a permanent reduction of fear may be realized through targeting the process of reconsolidation [5], [8]. Even though disrupting reconsolidation may thus be promising in dampening the impact of unwanted fears, one noticeable shortcoming is that the fear-reducing effects are thus far only demonstrated in the average organism (i.e., rodents - humans). Given that individual differences play a crucial role in the development of anxiety disorders [9], the value of research on normal fear learning and memory processes may be limited for the development of better treatments for anxiety disorders [10]. Hence, an important step towards advancing this basic research into clinical application would be to incorporate individual differences.

Processes of fear memory reconsolidation are traditionally investigated for a learned association between a visual or auditory stimulus (i.e., Conditioned Stimulus, CS) (e.g., pictures, tones) and a noxious event (i.e., Unconditioned Stimulus, US). Pavlovian fear conditioning indeed proved to be a valuable experimental model to test novel procedures that aim to dampen acquired fear responding. Not only the disruption of reconsolidation but also the traditional extinction procedure is well suited to diminish conditioned fear responding. Extinction training involves repeated unreinforced CS re-exposures and results in a new memory being formed that attenuates the behavioral responding to a feared stimulus [11]. Individual differences have already been demonstrated for fear extinction. Both patients with anxiety disorders and high trait anxious individuals showed impaired fear extinction as compared to normal healthy controls [12]–[16]. Also fear-conditioning studies in rodents showed that high anxiety rats were more resistant to fear extinction [17]–[20]. We asked ourselves whether disrupting fear memory reconsolidation - like fear extinction - is also less effective in high trait anxious individuals.

**Figure 1:**
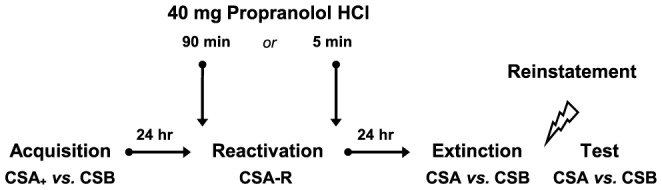
Schematic of the basic experimental procedure.

Here we addressed this issue by collapsing data from six previous fear conditioning studies on disrupting fear memory reconsolidation in humans [8], [21]–[24] - see Table 1 for the main features of the various experiments. In all of these studies testing included different phases across three subsequent days each separated by 24 h: fear acquisition on day 1 - memory reactivation on day 2 - and extinction and test on day 3 - see Fig. 1. Reconsolidation of the fear memory was manipulated by administering the β-adrenergic receptor antagonist *propranolol HCl* either before or after memory retrieval (i.e., day 2). Given that in our previous studies propranolol HCl only affected the startle fear responding - and the subjective feelings of anxiety in one experiment [24] - we here restricted our analysis to the eye blink startle reflex. Startle potentiation is considered a reliable and specific index of fear [25], which is directly connected with and modulated by the amygdalar complex [26]. Salivary alpha amylase and blood pressures were determined to ensure that the propranolol HCl manipulation exerted its intended physiological effect. Although each of the studies further differed procedurally in a number of ways (e.g., conditioned stimuli), an equal fear reduction was observed following the disruption of the reconsolidation process [8], [21]–[24] - see also the Results section. This offered the opportunity to collapse our six previous studies and to test whether high trait anxiety was related to less fear reduction following the disruption of memory reconsolidation by the noradrenergic β-blocker propranolol HCl. Less fear reduction would appear from more differential startle fear responding (i.e., CSa *vs.* CSb) at retention testing (i.e., day 3) in the participants with high trait anxiety as compared to those with low trait anxiety.

**Table 1:** Main features of our previous studies on fear memory reconsolidation.

Experiment	*n*	STAIT ± SD	Dependent Variables	CSs: IAPS Numbers	US in mA ± SD	Experimental Phases	Pill Administration
Kindt et al. 2009	20	32.9±5.1	FPS	CS1+ *vs.* CS2−:	14.1±3.3	**Day 1**: Acquisition	40 mg Propranolol HCl
			Online US Expectancy	IAPS: 1200–1201		8 CS1^+^ - 8 CS2	placebo-controlled.
						**Day 2**: Reactivation	90 minutes prior to
						1 CS1-R	memory reactivation.
						**Day 3**: Extinction	
						10 CS1 - 10 CS2	
						**Day 3**: Reinstatement	
						3 USs	
						**Day 3**: Test	
						5 CS1 - 5 CS2	
Soeter & Kindt 2010	20	33.2±7.9	FPS	CS1+ *vs.* CS2−:	15.9±9.9	**Day 1**: Acquisition	40 mg Propranolol HCl
			SCR	IAPS: 1200–1201		8 CS1^+^ - 8 CS2	placebo-controlled.
			Online US Expectancy			**Day 2**: Reactivation	90 minutes prior to
						1 CS1-R	memory reactivation.
						**Day 3**: Extinction	
						12 CS1 - 12 CS2	
						**Day 3**: Reinstatement	
						3 USs	
						**Day 3**: Test	
						1 CS1 - 1 CS2	
Soeter & Kindt 2011	15	36.1±7.2	FPS	CS1+ *vs.* CS2+ *vs.* CS3−:	19.7±8.7	**Day 1**: Acquisition	40 mg Propranolol HCl.
*Experiment I*			SCR	IAPS: 1200 - 6210 - 7009		5 CS1^+^ - 5 CS2 - 5 CS3	90 minutes prior to
			Online Distress			**Day 2**: Reactivation	memory reactivation.
			Retrospective US Expectancy			1 CS1-R	
						**Day 3**: Extinction	
						10 CS1 - 10 CS2 - 10 CS3	
						**Day 3**: Reinstatement	
						3 USs	
						**Day 3**: Test	
						5 CS1 - 5 CS2 - 5 CS3	
Soeter & Kindt 2012a	30	36.9±8.0	FPS	CS1+ *vs.* CS2+ *vs.* CS3−:	15.5±5.8	**Day 1**: Acquisition	*Experiment I:*
*Experiment I & II*	10	32.0±10.1	SCR	IAPS: 1200 - 6210 - 7009	17.3±5.6	5 CS1^+^ - 5 CS2 - 5 CS3	20 mg Yohimbine HCl
			Retrospective US Expectancy			**Day 2**: Reactivation	placebo-controlled.
						1 CS1-R	30 minutes prior to
						**Day 3**: Extinction	fear learning.
						10 CS1 - 10 CS2 - 10 CS3	AND
						**Day 3**: Reinstatement	40 mg Propranolol HCl.
						1 US	90 minutes prior to
						**Day 3**: Test	memory reactivation.
						1 CS1 - 1 CS2 - 1 CS3	*Experiment II:*
							40 mg Propranolol HCl.
							5 min *after* memory
							reactivation.
Soeter & Kindt 2012b	12	32.9±5.9	FPS	CS1+ *vs.* CS2−:	Described as a very unpleasant electric stimulus.	**Day 1**: Acquisition	40 mg Propranolol HCl
			Online Subjective Distress	IAPS: 1201 - 6200		3 CS1^+^ - 3 CS2	placebo-controlled.
			Retrospective US Expectancy			**Day 2**: Reactivation	5 min *after* memory
						1 CS1-R	reactivation.
						**Day 3**: Extinction	
						12 CS1 - 12 CS2	
						**Day 3**: Renewal	
						1 CS1 - 1 CS2	

FPS: Fear potentiated startle. SCR: Skin conductance responding. CS: Conditioned stimulus. US: Unconditioned stimulus. Note that in our current study the CSa stimulus always represents the CS1+ whereas the CSb stimulus always represents the unreinforced control stimulus, which either consists of the CS2 [Kindt et al. 2009, Soeter & Kindt 2010, 2012b] or the CS3 stimulus [Soeter & Kindt 2011, 2012a].

## Materials and Methods

### Participants

A total of 107 undergraduate students (25 men - 82 women) from the University of Amsterdam ranging in the age of 18 to 47 years (mean ± SD age - 20.6±3.3 years) participated in the various *propranolol HCl* conditions [8], [21]–[24]. All participants were assessed to be free from any current or previous medical or psychiatric condition that would contraindicate taking a single 40 mg dose of propranolol HCl (i.e., pregnancy - seizure disorder - respiratory disorder - cardiovascular disease - BP≤90-60 - diabetes - liver or kidney disorder - depression - psychosis). In order to eliminate individuals who might have difficulty with any temporary symptoms induced by the propranolol HCl manipulation, an additional exclusion criterion contained a score ≥26 on the Anxiety Sensitivity Index [27]. Note that trait anxiety is only marginally related to anxiety sensitivity [28]. Participants received either partial course credits or were paid a small amount for their participation in one of the experiments. Written informed consent was obtained from all participants and the ethical committee of the University of Amsterdam approved the studies.

### Apparatus and Measurements

#### Stimuli

In order to strengthen the fear association during acquisition a fear-relevant stimulus (i.e., a picture of a spider *or* gun: IAPS numbers 1200–1201 *or* 6200–6210) served as CSa^+^ [29]. A fear-(ir)relevant stimulus served as a control stimulus (i.e., CSb - a picture of a spider *or* gun *or* mug: IAPS number 1200–1201 *or* 6200*–*6210 *or* 7009). Pictures were 200 mm high and 270 mm wide and were presented in the middle of a black screen on a 19-in computer monitor. Whereas the CSa^+^ was followed by an US - or the threat of an US in one study [24] - during acquisition, the control stimulus (CSb) was not. Both the CSa and CSb stimuli were presented for 8 s. A startle probe was presented 7 s after CS onset and was followed by the US (CSa) 500 ms later. An electric stimulus of 2 ms that was delivered to the wrist of the non-preferred hand served as US. Delivery of the electric stimulus was controlled by a Digitimer DS7A constant current stimulator (Hertfordshire - UK) via a pair of Ag electrodes of 20 by 25 mm with a fixed inter-electrode mid-distance of 45 mm. A conductive gel (Signa - Parker) was applied between the electrodes and the skin.

#### Pharmacological Treatment

Propranolol HCl pills (40 mg) were prepared and blinded by the pharmacy (Huygens Apotheek - Voorburg - The Netherlands).

#### Fear Potentiated Startle

Conditioned fear responding was measured as potentiation of the eye-blink startle reflex to a loud noise by electromyography (EMG) of the right orbicularis oculi muscle. Loud noises (40 ms - 104 dB) were administered during each CS presentation and during inter-trial intervals (NA - noise alone). Two 7 mm Ag-AgCl electrodes filled with electrolyte gel were positioned approximately 1 cm under the pupil and 1 cm below the lateral canthus - a ground reference was placed either 3 cm below the orbicularis oculi pars orbitalis on an electrically neutral site or on the forehead [30]. All acoustic stimuli were delivered binaurally through headphones (Model MD-4600 - Compact Disc Digital Audio - Monacor). Eye-blink EMG activity was measured using a bundled pair of electrodes wires connected to a front-end amplifier with an input resistance of 10 MΩ and a bandwidth of DC-1500 Hz. A notch filter was set at 50 Hz to remove unwanted interference. Integration was handled by a true-RMS converter (i.e., contour follower) with a time constant of 25 ms. Integrated EMG signals were sampled at either 100 or 1000 Hz. Absolute peak amplitudes were identified over the period of 20–200 ms following probe onset and were recorded in microvolts.

#### Blood Pressure

Blood pressure was measured using an electronic sphygmomanometer (OMRON M4-I - Healthcare Europe BV - Hoofddorp - The Netherlands) with a cuff applied around the right upper arm.

#### Saliva Sampling

Salivary enzyme α-amylase (sAA) is a reliable indicator of noradrenergic activation [31]. Levels were assessed out of unstimulated saliva samples obtained using regular cotton Salivette sampling devices (Sarstedt - Nümbrecht - Germany) without chemical stimulants. Subjects were asked just to place the swab in their mouths for a 3 min period. After removal the Salivettes were stored at −25°C. To facilitate salivary sampling participants were instructed to refrain from exercise, caffeine and alcohol during the 12 hr before each session. Also, they were instructed to abstain from brushing their teeth for 1 hr and avoid food intake, drinking any beverages other than water and smoking for 2 hr before each session. Upon completion of the study, the samples were sent to Groningen for biochemical analysis (Universitair Medisch Centrum - Groningen - The Netherlands).

#### Subjective Assessments

State and trait anxiety were assessed with the State and Trait Anxiety Inventory [32]. Anxiety Sensitivity Index (ASI) [27] was used to assess one's tendency to respond fearfully to anxiety-related symptoms.

### Experimental Procedure

Participants were subjected to a(n) (instructed) differential fear conditioning procedure including several phases across three subsequent days each separated by 24 hr. During each session participants were seated in front of a computer screen at a distance of 50 cm in a sound-attenuated room. Each session began with a 1-min acclimation period consisting of 70 dB broadband noise - which continued throughout the session as background noise - followed by a habituation phase consisting of ten startle probes to reduce initial startle reactivity. Characteristics of the CSs, trial order, inter-trial intervals and startle probes as well as any instructions regarding ‘online’ ratings during memory reactivation and extinction-test were similar to acquisition.

#### Acquisition

Details of the various study procedures were explained and possible questions were answered. Participants were interviewed regarding their health and any medical or psychiatric conditions that would contraindicate taking a single 40 mg dose of propranolol HCl. Blood pressure was also measured. Once a participant was medically cleared, written informed consent was obtained, the ASI and STAI were administered and saliva samples were collected.

With the exception of the instructed fear learning experiment [24] - in which the US was described as causing a brief but *very unpleasant* sensation - the intensity of the US was determined after attachment of the different electrodes. Starting at an intensity of 1 mA the level of a 2-ms aversive electric stimulus delivered to the wrist of the non-preferred hand was gradually increased. Intensity of shock was individually set at a level defined by the participant as “uncomfortable - but not painful” and remained set to this intensity throughout the following days. After US selection participants were informed regarding the CSs. Depending on the study they were told that either one or two pictures would be followed by an electric stimulus in most of the cases. A second or third picture would never be followed by the US. They were instructed to learn to predict whether an electric stimulus would occur or not on the basis of the pictures. Apart from the experiments described in Soeter and Kindt - 2012*a* [23] participants were further required to either rate their expectancy of the electric stimulus or their level of distress during the presentation of each CS by shifting a cursor on a visual analog scale and push the left mouse button within 5 s following stimulus onset.

In the *acquisition* phase either one or two CSa^+^(s) and a control stimulus CSb were presented a number of times for 8 s: see Table 1. Startle probes were presented 7 s after CS onset and were followed by the US 500 ms later (CSa^+^s). A number of baseline startle probes were also presented alone (NA - noise alone). Order of trial types was randomized within blocks (i.e., CSa^+^(s) - CSb - NA). Inter-trial intervals varied between 15 - 20 - 25 s with a mean of 20 s. At the end of the first test session participants completed the STAI-s. Furthermore, they were explicitly instructed to remember what they had learned during acquisition.

#### Memory Reactivation

In order to substantiate consolidation of the fear memory a break of 24 hr after acquisition was inserted. Subsequent to the attachment of the electrodes the participants were instructed that the same pictures would be presented and - to ascertain that the three test sessions were part of one experiment - they were asked to remember what they had learned during acquisition. In the *memory reactivation* phase only a single CSa-R was presented for 8 s followed by a startle probe presented alone.

All of the participants received single or double blind an oral dose of 40 mg of propranolol HCl either 90 min *before* reactivation of the memory or 5 min *after* memory reactivation (i.e., CSa-R) [33]. STAI-s was filled out both before and upon completion of the experiment. Furthermore - at these time-points - blood pressure and saliva samples were collected.

#### Extinction - Reinstatement Testing

Upon arriving at the experimental site, blood pressure and saliva samples were again collected and the STAI-s was completed. After attachment of the electrodes participants were simply informed that the same pictures provided during acquisition would be presented. In the *extinction* phase participants were exposed to the feared CSa and safe CSb stimuli in the absence of the electric stimulus (US). A number of startle probes were again presented alone (NA). In all but one of the experiments we presented either *one* or *three* unsignaled reminder shock(s) after the extinction procedure in order to reinstate the expression of the original fear memory. Timing between the last extinction trial and the (first) reinstating US was 19 s. Following the unsignaled US(s) participants were again presented with one or more CSa, CSb and NA trials (i.e., reinstatement testing). Timing between the (last) reinstating US and reinstatement testing was 18 s. At the conclusion of the experiment the participants completed the STAI-s.

### Statistical Analysis

We collapsed the data from six studies into one overall data set [8], [21]–[24]. Given that the amount of conditioned stimuli and the number of stimulus presentations during acquisition and extinction learning differed between the various studies, we only included (a) the reactivated fear conditioned stimulus (CSa) as well as the unreinforced control stimulus (CSb) and (b) the *first* and the *last* trial of each of the different experimental phases. We performed a mixed analysis of variance for repeated measures with study as between-subjects factor and stimulus (i.e., type of CS) and trial (i.e., stimulus presentation) as within-subjects factors to verify whether the reconsolidation of the fear memory was equally affected by the propranolol HCl manipulation in the various studies [8], [21]–[24]. Next we performed a two-step hierarchical regression with “fear-reducing effect of disrupting memory reconsolidation” - which was calculated by subtracting the differential startle responding on the last trial of fear acquisition from the differential startle responding on the first trial of extinction learning - as dependent variable and trait anxiety scores entered in the first step. In step two we entered (a) state anxiety scores obtained before memory reactivation as well as percent changes in (b) systolic and (c) diastolic blood pressures and (d) salivary alpha amylase following the propranolol HCl manipulation and (e) the Body Mass Index (BMI) of the participants. We then selected participants that were either *high* or *low* on their trait anxiety scores [32] and performed various ANOVAs and *t* tests for determining the fear-reducing effects within groups. Missing data were excluded from the analysis. Significance was set at *p*<0.05.

## Results

### Comparisons of the various Propranolol HCl conditions

Analysis of variance showed a comparable fear learning on day 1 in our six studies as is demonstrated by a significant increase of the differential startle fear responding from the first acquisition trial to the last trial of acquisition [CSa *vs.* CSb; stimulus×trial, *F*_1,99_ = 69.46, *p*<0.001, *η*_p_^2^ = .41; stimulus×trial×study, *F*_5,99_<1.80]. Moreover, the propranolol HCl groups expressed similar levels of differential startle potentiation during memory reactivation (i.e., day 2) [CSa-R *vs.* NA; stimulus×study, *F*_5,100_<1]. Startle fear responding remained relatively stable from the last trial of acquisition to memory reactivation which further demonstrates that the acquired fear memory was equally well consolidated in the various propranolol HCl groups [CSa *vs.* NA; stimulus×trial×study, *F*_5,100_<1]. Administration of the β-blocker similarly decreased the differential startle fear responding from the last trial of acquisition to the first extinction trial 48 hr later (i.e., day 3) [CSa *vs.* CSb; stimulus×trial, *F*_1,99_ = 72.84, *p*<0.001, *η*_p_^2^ = .42; stimulus×trial×study, *F*_5,99_<1.93]. Furthermore, we did not observe a differential change in startle fear responding from the first extinction trial to the last trial of extinction learning in any of the propranolol HCl groups [CSa *vs.* CSb; stimulus×trial, *F*_1,99_<1; stimulus×trials×study, *F*_5,99_<1]. Exposure to the *reminder shocks* also failed to uncover any differential startle fear responding in all of the propranolol HCl groups [CSa *vs.* CSb; stimulus×trial *F*_1,88_<2.54; stimulus×trial×study, *F*_4, 88_<1]. Together these findings indicate that the reconsolidation of the CSa fear memory was equally disrupted by the propranolol HCl manipulation in the various experiments.

### Trait anxiety uniquely predicts the fear-reducing effects of disrupting memory reconsolidation

Trait anxiety scores ranged from 21 to 55 with a mean of 34.5 (SD = 7.8). Furthermore, we observed a significant decrease in systolic and diastolic BP [moment, *t*_106_ = 19.07, *p*<0.001, two-tailed; *t*_106_ = 10.58, *p*<0.001, two-tailed, respectively] (see also Table 2) as well as alpha amylase [moment, *t*_66_ = 4.08, *p*<0.001, two-tailed] following the propranolol HCl administration. This indicates that the pill manipulation exerted its intended physiological effect. Blood pressure and salivary alpha amylase again returned to baseline levels at retention testing (i.e., day 3) given that we observed no effect of propranolol HCl on the course of the systolic and diastolic BP [day 1 *vs.* day 3; moment, *t*s_106_<1.48] as well as sAA levels [day 1 *vs.* day 3; moment, *t*_66_<1].

**Table 2:** Mean values (SD) of the systolic and diastolic blood pressure in mmHg and amylase level in U-ml for the combined propranolol HCl conditions.

	*Day 1*	Pre Pill Intake *Day 2*	Post Pill Intake *Day 2*	*Day 3*
**Systolic BP**	127.5 (SD = 13.4)	128.1 (SD = 11.3)	111.5 (SD = 8.9)	126.5 (SD = 12.5)
**Diastolic BP**	74.5 (SD = 7.0)	73.1 (SD = 8.4)	67.3 (SD = 7.1)	73.4 (SD = 7.3)
**sAA Level**	71.7 (SD = 84.1)	81.1 (SD = 97.5)	32.7 (SD = 33.6)	74.2 (SD = 92.3)

**Figure 2:**
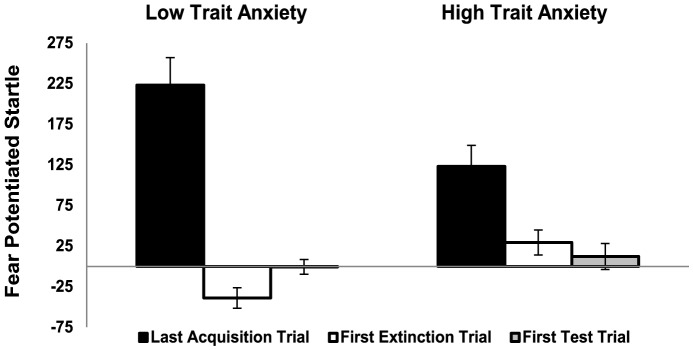
Trait anxiety determines the fear-reducing effects of disrupting memory reconsolidation. Mean startle amplitudes in microvolts during the last trial of acquisition, the first extinction trial and the first test trial for the Low Trait Anxiety and High Trait Anxiety groups. Startle potentiation was calculated by subtracting the startle responding to the control CSb stimulus from the startle responding to the fear conditioned CSa stimulus during the corresponding test trial. Error bars represent SEM.

A hierarchical regression analysis showed that neither the reduction in BP and sAA following the propranolol HCl manipulation (i.e., day 2) nor the BMI and the anxiety state before memory retrieval were related to the fear reducing effects of disrupting reconsolidation - see Table 3. Instead trait anxiety uniquely predicted the effectiveness of targeting the process of reconsolidation by propranolol HCl: higher trait anxiety scores resulted in less fear reduction at retention testing (i.e., day 3) - see also Table 3. We next selected participants on the basis of their trait anxiety scores. High trait anxiety was defined as scoring within the upper quartile of the STAI-t (i.e., STAI-t scores >40: *n* = 26, M = 45.5, SD = 4.2) and low trait anxiety as scoring within the lowest quartile of this inventory (i.e., STAI-t scores <28: *n* = 28, M = 26.1, SD = 2.2). Note that the trait anxiety scores of the HTA group fall within the range of a high anxiety norm group consisting of students and 1 SD below the mean norm scores for psychiatric patients [34]. For the LTA group the mean trait anxiety scores were 1 SD below the mean of a student population [34]. Selecting participants based on their trait anxiety scores indeed revealed a significant difference in the amount of fear reduction at retention test between groups [CSa *vs.* CSb; stimulus×trial×group, *F*_1,51_ = 13.54, *p* = 0.001, *η*_p_^2^ = .21]. That is the reduction in startle fear responding from the last trial of acquisition to the first extinction trial 48 hr later was more pronounced in the *low* trait anxiety group [CSa *vs.* CSb; stimulus×trial, *F*_1,27_ = 58.81, *p*<0.001, *η*_p_^2^ = .69;] than in the *high* trait anxiety group [CSa *vs.* CSb; stimulus×trial, *F*_1,24_ = 10.53, *p*<0.01, *η*_p_^2^ = .31]. In the *high* trait anxiety group there was still some fear left (i.e., CSa>CSb) on the first trial of extinction learning [*t*_24_ = 1.94, *p*<0.05, one-tailed] (see Fig. 2), whereas the startle fear responding was even potentiated in the opposite direction (i.e., CSa<CSb) in the *low* trait anxiety group [*t*_24_ = −3.09, *p*<0.01, two-tailed] (see also Fig. 2). We further observed no relation between trait anxiety and the return of fear following the *reminder shocks* [i.e., *p*>.30].

**Table 3:** Results from the Hierarchical Multiple Regression analyses.

	*t*	*B*
*Step I*		
STAI-t	2.44	.26*
*Step II*		
STAI-t	2.09	.28*
STAI-s	<1	.06
Systolic BP	<1	.04
Diastolic BP	1.17	.12
sAA Level	<1	.005
BMI	1.15	.13

Note that R^2^ = .09 for Step I (*p*<0.05) and Δ R^2^ = .03 for Step II (*p*>0.05).

* *p*<0.05.

## Discussion

Here we show that trait anxiety impairs the fear-reducing effects of disrupting memory reconsolidation: the higher the trait anxiety, the less fear reduction. We further show that (a) the anxiety state of the participants prior to memory reactivation and (b) the physiological effects caused by the drug propranolol HCl - i.e., reduction in blood pressure and salivary alpha amylase - are not related to the amount of fear-reduction. Although propranolol HCl exerted its normal physiological effects [8], [21]–[24], the current noradrenergic blockade may still not have been optimal for the high trait anxiety group. As a result, the restabilization of the fear memory may not have been fully disrupted. Higher dosages of propranolol HCl may thus be required for better treatment effects in high trait anxious individuals. Another possibility is that high trait anxiety demands a different reactivation protocol in order to induce fear memory destabilization. An important function of reconsolidation might be to maintain memory relevance in guiding future behavior [35]–[36]. Labilization and subsequent reconsolidation do indeed not necessarily occur when a memory is reactivated but only when there is something to be learned during memory retrieval [36]–[39]. A violation based upon prior learning is supposed to be a necessary condition for reconsolidation such that the actual outcomes of an event do not match with what was predicted based upon prior experiences [24], [37]–[41]. Considering that high trait anxious individuals often utilize a better-safe-than-sorry strategy in ambiguous situations [42], it may be suggested that the single unreinforced reactivation trial was insufficient for an optimal destabilization of the fear memory trace following the partial reinforcement scheme during fear acquisition. Note that we did find a clear - but less pronounced - fear reduction in the high trait anxiety group and that trait anxiety was not related to the return of fear following the reminder shocks. Hence the current findings do *not* suggest that high trait anxiety is a *boundary condition* for reconsolidation but rather that the current protocol of one retrieval trial and 40 mg of propranolol HCl was not optimal for dampening the expression of the previously formed fear memory in individuals with high trait anxiety.

Our finding of limited fear reduction in individuals who are vulnerable to develop an anxiety disorder clearly shows that we cannot simply translate fundamental animal and human research on fear memory reconsolidation to clinical practice. This is particularly important when considering that the trait anxiety scores in the present aggregated sample were still lower than those of clinical populations [32], [34], [43]. But given its superiority over fear extinction [8], [21]–[24], targeting the process of reconsolidation still points to a very promising therapeutic tool for providing long-term cure for patients suffering from anxiety disorders. Further research is needed for addressing the issue of individual differences in the malleability of emotional memory.
